# Construction of microneedle of *Atractylodes macrocephala Rhizoma* aqueous extract and effect on mammary gland hyperplasia based on intestinal flora

**DOI:** 10.3389/fendo.2023.1158318

**Published:** 2023-02-28

**Authors:** Yang Ping, Qi Gao, Changxu Li, Yan Wang, Yuliang Wang, Shuo Li, Mingjing Qiu, Linqian Zhang, Ailing Tu, Yu Tian, Hong Zhao

**Affiliations:** College of Pharmacy, Jiamusi University, Jiamusi, Heilongjiang, China

**Keywords:** aqueous extract, microneedle, mammary gland hyperplasia, intestinal flora, *Atractylodes macrocephala Rhizoma*

## Abstract

**Background:**

A microneedle patch loaded with *Atractylodes macrocephala Rhizoma* water extract was prepared for the treatment of mammary gland hyperplasia. To explore the relationship between Mammary gland hyperplasia and intestinal flora.

**Materials and methods:**

Preparation of the microneedle patch by micromolding method, the prescription of the microneedle was optimized by the Box-Behnken Design response surface test, and the micro-morphology, penetration, toughness, and brittleness were investigated. *In vitro* release of drug-loaded microneedles was measured by diffusion cell method. The rat model of mammary gland hyperplasia was prepared by the combination of estradiol benzoate-progesterone, and the microneedle patch of *Atractylodes macrocephala Rhizoma* aqueous extract was used for intervention treatment. The change of levels in E_2_, P, and PRL in rat serum was determined. The intestinal contents of rats were collected and the changes in intestinal flora in MGH rats were analyzed by 16s rRNA high-throughput sequencing.

**Results:**

The optimized microneedle formula is a PVA concentration of 6.0%, HA concentration of 15.5%, and PVPK30 concentration of 16.0%. The prepared microneedle tip loaded with *Atractylodes macrocephala Rhizoma* aqueous extract has complete, sharp, and no bubbles and the needle rate of the microneedle array is in the range of 95%~100%. The bending rate of the microneedle is about 12.7%, and it has good flexibility, and the microneedle can puncture 4 layers of ParafilmⓇ membrane smoothly, and the puncture rate is more than 96%. The *in vitro* release of the microneedle was characterized by rapid release. The results of animal experiments showed that *Atractylodes macrocephala Rhizoma* aqueous extract microneedle patch could significantly reduce the E_2_ level, significantly reduce the PRL level, and significantly increase the P level. At the same time, it can regulate the abundance and diversity of intestinal flora in MGH rats, improve the intestinal flora disorder caused by mammary gland hyperplasia, and balance the community structure.

**Conclusion:**

The prepared microneedle containing *Atractylodes macrocephala Rhizoma* aqueous extract has good toughness and brittle strength, can penetrate the skin and enter the dermis, and effectively deliver drugs to play a role in the treatment of mammary gland hyperplasia.

## 1 Introduction

Mammary gland hyperplasia (MGH) is a non-inflammatory and non-tumor chronic proliferative disease, mainly characterized by breast lumps and breast pain. At present, hormone inhibition and local resection are commonly used in western medicine. Hormone inhibition can only relieve symptoms and has a large side effect. Local resection is not easy for patients to accept. In recent years, traditional Chinese medicine (TCM) has had the advantages of good compliance and fewer adverse reactions in treating MGH, which can effectively improve the symptoms of patients. The patients were satisfied with the external treatment of TCM. External treatment of percutaneous drug delivery can avoid the “first-pass effect” of the liver and damage to the gastrointestinal tract so that the drug can enter the blood circulation through the skin and play a systemic role. At the same time, it can effectively improve bioavailability, maintain stable and lasting blood drug concentrations, and have other advantages.

Microneedle is a kind of transdermal drug delivery. The extremely delicate micro needle cluster made by micro-manufacturing technology can penetrate the cuticle of the skin, forming temporary aqueous micropores on the skin surface, so that the drug can diffuse into the skin through the micropores, thus exerting the efficacy to achieve microcirculation. Soluble microneedles, also known as dissolving microneedles (DMN) (1, 2), are polymer microneedles that encapsulate drugs in biodegradable polymer materials. When DMN is inserted into the skin, the polymer degrades spontaneously to release drugs. The drug release rate is mainly related to the properties of drugs and dosage forms. Therefore, DMN can achieve local or systemic therapeutic effects in the short or long term. At the same time, the tiny pores left by DMN on the skin surface can heal themselves in a short time, reducing the risk of infection caused by the fracture of the solid tip in the skin, and realizing a safe and painless drug delivery method in a real sense.

**Figure 1:**
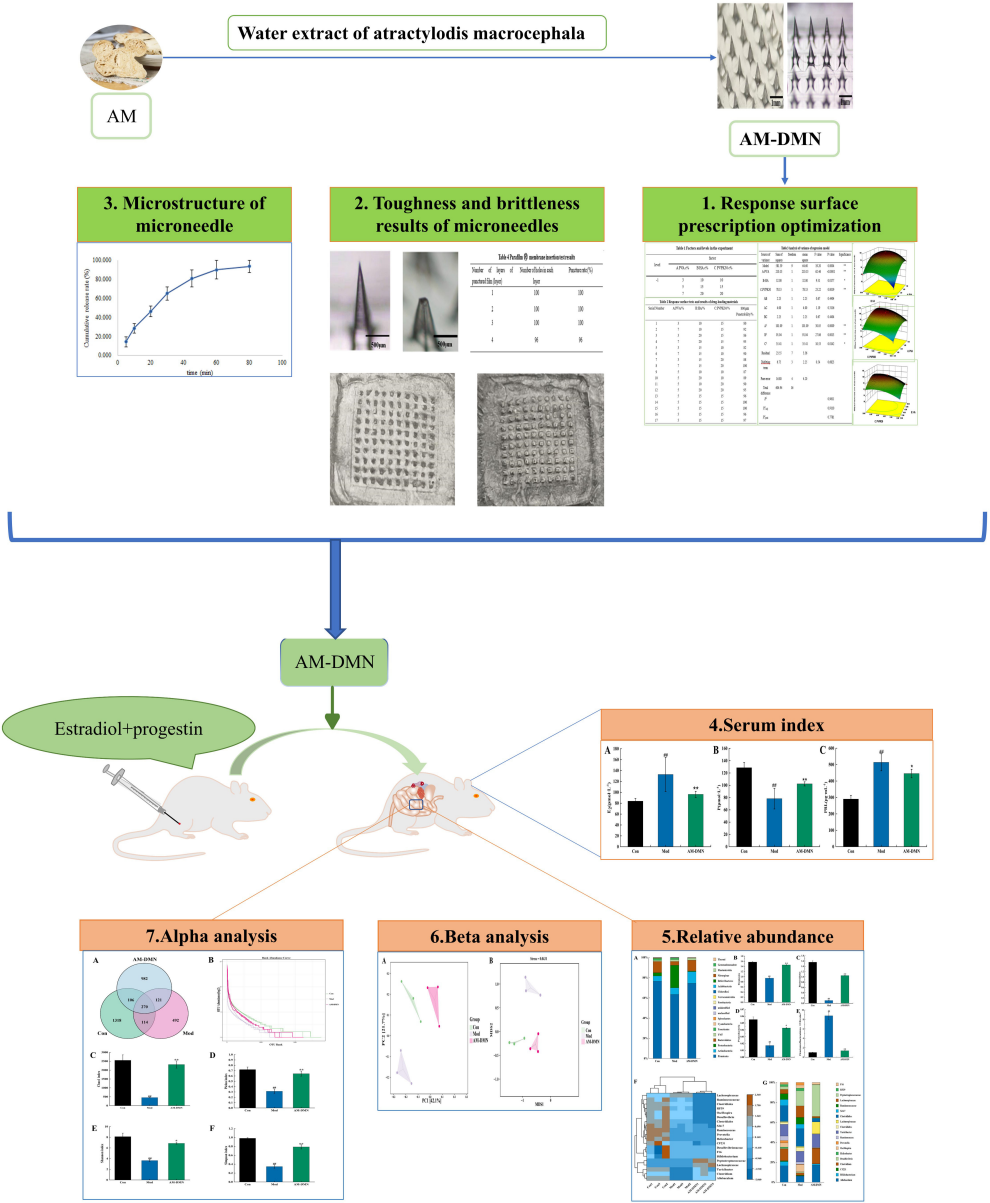
Experimental flow graph.

Modern medical investigation shows that MGH is caused by endocrine disorders. The mammary gland is a target organ of sexual hormones, it regulates hyperplasia of breast tissue and the cycle through by regulating serum hormones, and hypothalamic-pituitary-gonads (3). As early as 1883, it was proposed that the MGH may be associated with sexual hormones. It was first proposed in 1947 that the disorder of estrogen and progestogen was the cause of the MGH (4). However, the endocrine imbalance can affect the intestinal flora composition and directly or indirectly change bacterial physiology and independent gene expression (5). Estrogen imbalance, as an important sign of endocrine disorder, has also been proven to significantly affect the changes in intestinal microbiota (6). A large number of studies have proved that TCM can regulate the imbalance of intestinal microbiota significantly, Cuiru Li et al. showed that QWBZP crude polysaccharide helped to restore the diversity, relative abundance, and community structure of intestinal mucosal bacteria to a certain extent (7). Xiaoya Li et al. explored the role of intestinal contents microbiota in the regulation of adverse effects caused by high-fat diet by DO from the perspective of intestinal microecology. And demonstrated that the mechanism of DO against a high-fat diet diseases might be attributed to the inhibition of Ruminococcus and Oscillospira, leading to a promotion in the state of host health (8, 9). Moreover, intestinal flora coincides with the “spleen and stomach” theory of TCM. TCM can promote the growth of beneficial bacteria, inhibit the excessive production of harmful bacteria, balance the number of beneficial bacteria and pathogenic bacteria, and maintain a healthy intestinal environment (10). The composition and quantity of intestinal flora are in balance under normal conditions. Once the balance is broken by factors such as intestinal pH value, mental pressure, eating habits, and antibiotic use, it will cause metabolic obstacles, metabolic disorders, and even other related diseases to some extent (11–16). The dried rhizome of the plant *Atractylodes macrocephala* Koidz is useful for drying and moistening water, nourishing the stomach, and strengthening the spleen. *Atractylodes macrocephala Rhizoma* has been reported to include amino acids, polysaccharides, and a range of volatile components. It also has been proven to have pharmacological effects, such as controlling intestinal flora and enhancing digestive function (17). In order to provide theoretical support for the treatment of MGH from the perspective of intestinal microecology and provide an experimental basis for the full exploitation of the medicinal value of Atractylodes macrocephala Rhizoma aqueous extract (AM-ae), this study will prepare a microneedle patch for the treatment of MGH rats using AM-ae. It will also analyze the effect of AM-ae on the intestinal microbiota of MGH rats by 16S RNA high-throughput sequencing. **Figure 1** depicts the concept for the research.

## 2 Materials and methods

### 2.1 Drugs and materials

Polyvinyl alcohol 224 (purchased from Shanghai McLean Biochemical Technology Co., Ltd., CAS No. 9002-89-5; alcoholysis degree: 87.0-89.0 mol%; viscosity: 40.0-48.0 mPa. s); Hyaluronic acid (purchased from Shanghai McLean Biochemical Technology Co., Ltd., batch number: C12698745; molecular weight: 200000-400000); Polyvinylpyrrolidone K30 (purchased from Shanghai Aladdin Biochemical Technology Co., Ltd., batch number: H2112016; molecular weight: 40000, high purity); Water extract of *Atractylodes macrocephala Rhizoma* (self-made in the laboratory); Absolute ethanol (purchased from Tianjin Kaitong Chemical Reagent Co., Ltd.); Parafilm M ^®^ (Bemis Company, Inc).

Eighteen SPF-grade female non-pregnant SD rats (200 ± 20 g) were purchased from Changchun Lewis Laboratory Animal Technology Co., Ltd (Licence No.: SCXK(Ji)-2018-0007) and housed in SPF-grade animal rooms. The temperature and humidity in the rearing room were controlled, temperature: 24 ± 2°C, humidity: 60 ± 5%. The light and dark cycle was 12/12 h. All experimental procedures involving animals were approved by the Animal Ethics Committee of the Animal Experimentation Centre of Jiamusi University.

Purchases were made from Shanghai Quanyu Biotechnology (Zhumadian) Animal Pharmaceutical Co., Ltd. for estradiol benzoate and progesterone injection. Estradiol (E_2_), progesterone (P), and prolactin (PRL) kit was purchased from Jiangsu enzyme immunity Industrial Co., Ltd.

### 2.2 Instruments and equipment

FA2004N Electronic Analytical Balance (Shanghai Hengping Scientific Instrument Co., Ltd.); PDMS mold (Micropoint Technologies PTE LTD, Singapore); Xiangyi TDZ5-WS desktop low-speed centrifuge (Hunan Xiangyi Laboratory Instrument Development Co., Ltd.); Air blast dryer (Shanghai Hengping Scientific Instrument Co., Ltd.); Electron Microscope (Shanghai Hengping Scientific Instrument Co., Ltd.).

### 2.3 Preparation method of microneedle

Weigh the appropriate amount of polyvinyl alcohol 224 (PVA), hyaluronic acid (HA), polyvinylpyrrolidone K30 (PVPK30), and add an appropriate amount of double steaming water to dissolve them, and put them in the refrigerator to swell for 30~40 min. The three kinds of drug solutions are proportional to 1:1:1 ~ 1:1: 5 Mix well (solution 1), take the extract of *Atractylodes macrocephala Rhizoma* water with the drug loading capacity of 0.3%~2.5%, dissolve in a certain amount of anhydrous ethanol (solution 2), mix the solutions 1 and 2 evenly, drop them into the PDMS mold, and centrifuge at 2700~3300 r/min for 8~12min. After removal, it was dried for 50~70min in a drying box at 30°, then the backing layer solution was drip-added to the PDMS mold, centrifuged in a centrifuge at 3000r/min for 8~12min, and dried in a drying box at 55~65° for 50~70min. The drug-loaded microneedle array is obtained after demoulding.

### 2.4 Response surface prescription optimization

Based on single factor test, three factors, namely PVA concentration (A), HA concentration (B), and PVPK30 concentration (C), were selected as independent variables with the microneedle penetration rate of 800μm was taken as the response value, and the response surface test is designed according to the principle of Box Behnken Design (BBD). The optimization analysis was carried out according to the test results, and the formulation composition response surface method test design is shown in **Table 1**.

**Table 1:** Factors and levels in the experiment.

Levels	Factors		
	A:PVA c%	B:HA c%	C:PVPK30 c%
-1	3	10	10
0	5	15	15
1	7	20	20

### 2.5 Characterization of AM-DMN

The morphology of microneedle arrays was observed by electron microscope. The toughness of the microneedles was evaluated by the compression performance test: the microneedle tip was placed down on a smooth plane, a plane was added on the back of the microneedle to make it stressed evenly, and then place a certain weight of weight, and gradually increase the weight of the weight. Remove the weight after every 1-2 minutes of action, and the changes in the microneedle tip and height were observed.

The brittleness of the microneedles is characterized by the mechanical strength of the microneedles. The mechanical strength of the prepared microneedles can be evaluated by the ParafilmⓇ membrane insertion experiment. Stack 4 pieces of Parafilm Ⓡ film together, with a thickness of about 0.5~1 mm was placed on the foam. The microneedles were inserted into the ParafilmⓇ membrane and pressed for about 30~60 s. After the microneedles were removed, the number of layers of the membrane broken by the microneedles and the number of holes in each membrane was recorded.

The microneedle patch was placed on the dialysis membrane by diffusion cell method, and the receiving cell contained 10 mL PBS. The samples were taken at 37° for 1, 2, 5, 30, 60, and 300 min. The content of the aqueous extract of *Atractylodes macrocephala Rhizoma* in a microneedle patch was determined by the UPLC method, and the drug release performance of the microneedle patch was evaluated.

### 2.6 Experimental approach

A total of 18 female SD rats were chosen, acclimated, and fed for a week. Six rats from each group were randomly assigned to one of three groups: an AM-DMN group, a model control group (Mod), and a blank control group (Con). The rats in each group were injected with estradiol benzoate 0.5 mg-kg^-1^-D^-1^ intramuscularly for 25 days, followed by progesterone 5 mg-kg^-1^-D^-1^ intramuscularly for 5 days, except for the Con group, which received an identical dose of saline. The AM-DMN group received treatment with a medication microneedle patch after successful modeling, whereas the Con and Mod groups received treatment with a blank microneedle patch. These treatments were alternated every two days for a total of 21 days (18).

### 2.7 Collection and analysis of serum samples

Rats in each group fasted for 12 hours after the last dose. The rats were weighed and anesthetized intraperitoneally and injected with 2% sodium pentobarbital. The execution was performed using the cervical dislocation method. To isolate the serum, blood was taken from the abdominal aorta and stored in a procoagulant tube for 20 min. ELISA was used to determine serum E_2_, P, and PRL levels (18).

### 2.8 Collection of samples of intestinal cecum contents

After the death of the rats, the contents of the cecum were extracted using sterile forceps, placed in 5mL sterile EP tubes, numbered and weighed, then immediately immersed in liquid nitrogen under sterile conditions. After collection, the samples were transferred to -80°C for storage (18).

### 2.9 16S rRNA gene high-throughput sequencing

The cecum’s contents were taken. Following the instructions on the DNA extraction kit exactly, the total DNA content was extracted. DNA was measured with a nanodrop spectrophotometer (DNA quantitative analysis), and electrophoresis was used to determine the purity and concentration of the extracted genomic DNA. The 16S rDNA V3–V4 variable region was used for amplification, and the Quantit PicoGreen dsDNA analysis kit was used for fluorescence quantification. Samples were blended in the proper ratios for further fluorescence measurement. The contents of the rat cecum were then double-end sequenced using Illumina’s NovaSeq 6000 sequencer (18).

### 2.10 Bioinformatics and statistical analysis

Using the QIME2 DADA2 program, non-repeat sequence OTU clustering was carried out at a 97% similarity. The Greenenes database (version 13.8) was used to annotate the classification of species. To determine species richness and diversity, alpha diversity indices (Chao1, ACE, Simpson, and Shannon indices) were computed. Additionally, the beta diversity of the gut microbiota in various samples was examined, and this can determine the similarities and variations in the community composition of the various samples (or subgroups).

Data were analyzed using SPSS 26.0 statistical software. All data in the experiment are expressed as mean ± standard deviation (X ± s). One-way ANOVA was used for comparison between groups. p<0.05 was considered statistically significant. Drawings were expected to be completed using R 4.1.3 and GraphPad Prism 8 software.

## 3 Results

### 3.1 Prescription optimization of microneedles

With the penetration rate of 800μm microneedle as the index, the drug-carrying material test results in **Table 2** were analyzed by using Design Expert 8.0.6 software, and the regression equation of the penetration rate of 800μm microneedle was obtained as follows: 800μm=97.80+5.13A+2.00 B+3.13 C-0.75 AB+1.00 AC+0.75 BC-4.90 A^2^-4.65 B^2^-2.90 C^2^.

**Table 2:** Response surface tests and results of drug-loading materials.

Serial Number	A:PVA/%	B:HA/%	C:PVPK30/%	Penetrability/%
1	3	10	15	80
2	7	10	15	92
3	3	20	15	86
4	7	20	15	95
5	3	15	10	82
6	7	15	10	90
7	3	15	20	88
8	7	15	20	100
9	5	10	10	87
10	5	20	10	89
11	5	10	20	90
12	5	20	20	95
13	5	15	15	96
14	5	15	15	100
15	5	15	15	100
16	5	15	15	96
17	5	15	15	97

The software Design Expert 8.0.6 was used to establish the model, and the multiple quadratic regression response surface model of 800μm microneedle penetration rate was obtained. The multiple linear regression and binomial fitting analysis were conducted on the test results of microneedle prescription materials to verify the significance of the regression model and factors. The results of ANOVA are shown in **Table 3**.

**Table 3:** Analysis of variance of regression model.

Source of variance	Sum of squares	freedom	mean square	F value	*p* value	Significance
Model	581.39	9	64.60	19.20	0.0004	**
A-PVA	210.13	1	210.13	62.46	<0.0001	**
B-HA	32.00	1	32.00	9.51	0.0177	*
C-PVPK30	78.13	1	78.13	23.22	0.0019	**
AB	2.25	1	2.25	0.67	0.4404	
AC	4.00	1	4.00	1.19	0.3116	
BC	2.25	1	2.25	0.67	0.4404	
A^2^	101.09	1	101.09	30.05	0.0009	**
B^2^	91.04	1	91.04	27.06	0.0013	**
C^2^	35.41	1	35.41	10.53	0.0142	*
Residual	23.55	7	3.36			
Disfitting term	6.75	3	2.25	0.54	0.6823	
Pure error	16.80	4	4.20			
Total difference	604.94	16				
*R^2^*					0.9611	
*R^2^*_Adj_					0.9110	
*R^2^*_pred_					0.7781	

p ≤ 0.01 indicates that the factor has a very significant effect on the response value (**), P ≤ 0.05 indicates that the factor has a significant effect on the response value (*).

It can be seen from **Table 3** that F=19.20, p=0.0004 in the established regression model, indicating that the difference in the regression model is extremely significant; The p of the misfitting term is 0.6823>0.05, and the model difference is not significant, indicating that the equation is reliable; The regression coefficient *R* ^2 =^ 96.11%>85%, indicating that the equation is well fitted. The regression equation can be used to replace the real point of the test to describe the relationship between each variable and the response value. The correction coefficient $R_{\text{Adj}}^{2}$ = 0.9110 indicates that the model can explain the change in the penetration rate of microneedles of 91.10% 800μm. The data in **Table 3** show that the test design is reliable with small errors, which is suitable for the actual situation and can be used to analyze and predict the results of the microneedle preparation test.

It can be seen from the *p*-value in **Table 3** that the PVA concentration (A) and PVPK30 concentration (C) in the primary item have a very significant impact on the preparation of microneedles, and the HA concentration (B) has a significant impact; In the quadratic term, A^2^ and B^2^ have extremely significant effects on the preparation of microneedles, and C^2^ has significant effects on the preparation of microneedles. Among the interaction terms, AB, AC, and BC had no significant influence on the preparation of microneedles. According to the value of F in **Table 3**, it can be concluded that the influence of the three factors on the preparation of microneedles is PVA concentration (A)>PVPK30 concentration (C)>HA concentration (B).

**Figure 2:**
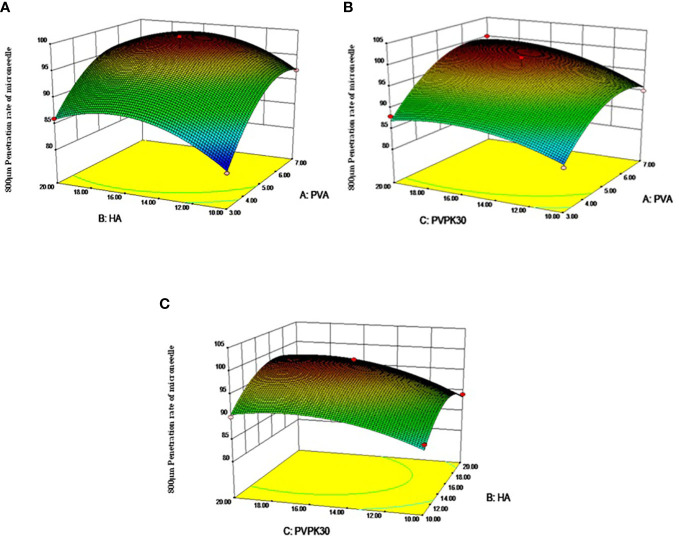
Response surface diagram and contour diagram of the interaction of various factors of microneedle prescription on the influence of microneedle preparation. **(A)** PVA and HA interactive response surface, **(B)** PVA and PVPK30 interactive response surface, **(C)** PVPK30 and HA interactive response surface)

As shown in **Figure 2 (A1, B1, C1)** for the response surface and contour map of interaction effects of microneedle materials PVA, HA, and PVPK30 created by the response surface regression model.

Validation experiment: Solve the regression fitting equation of the microneedle material, and obtain the best preparation conditions: A=6.08, B=15.74, C=16.27. Under this condition, the predicted penetration rate of the microneedle is 100.045%. According to the experimental and practical feasibility, the conditions for preparing microneedles by adjusting the modified drug loading material are as follows: PVA concentration was 6.0%, HA concentration was 15.5%, and PVPK30 concentration was 16.0%. Under these conditions, the penetration rate of microneedles was 97.5%, and the relative error of the predicted value of the model was only 2.54% (< 5%), indicating that the response surface method optimized the conditions for the preparation of microneedles, the preparation scheme parameters obtained were accurate and reliable, and had certain application value.

### 3.2 Characterization of AM-DMN

**Figure 3:**
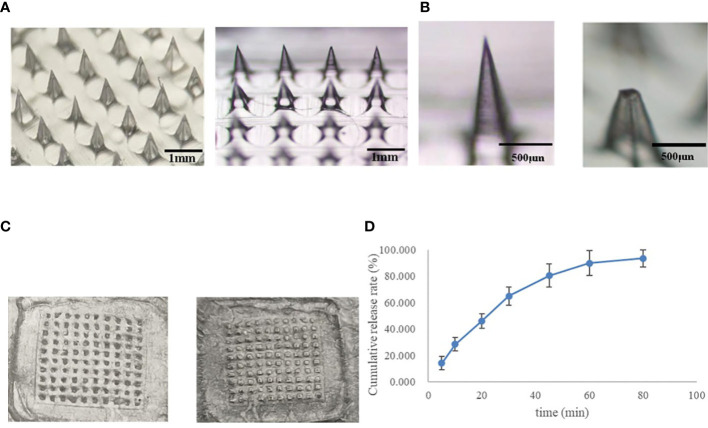
Microneedle characterization results **(A)**. Microneedle, **(B)** Appearance of microneedle under microscope (Left: 800 μ m. Right: 696 ± 2.5 μ m), **(C)** Micropuncture into 4 layers ParafilmⓇ membrane (Left: front, right: reverse), **(D)** Microneedle release *in vitro* (n=3)).

The morphology of the whole micro-needle array is a pyramid; The needle tip is complete and sharp, without bending, broken needle and bubble; The needle output rate of the microneedle array is in the range of 95%~100%; The micro-needle tip and the backing layer have good compatibility, the tip and the backing layer are not separated, and the backing layer is flat without bubbles. As shown in **Figure 3A**.

With the weight of 100g, 200g, 300g, 400g, and 500g increasing in turn, the tip of the microneedle will slightly bend. When the weight of the weight is 500g, the height of the microneedle will change from 800 μ M reduced to 696 ± 2.5 μ m. The bending rate of the microneedle is about 12.7% (**Figure 3B**), which shows that the prepared microneedle has good flexibility.

As shown in **Table 4** and **Figure 3C**, the prepared height is 800μm, the microneedle can puncture 4 layers of ParafilmⓇmembrane smoothly, and the puncture rate is more than 96%. The thickness of the cuticle of skin and active epidermis is about 100 μm. The thickness of the dermis is about 3-5 mm. Therefore, the prepared microneedle has good mechanical strength (brittleness) and can successfully penetrate the dermis to form a microchannel on the skin surface.

**Table 4:** Parafilm Ⓡ membrane insertion test results.

Number of layers of punctured film	Number of holes in each layer	Puncture rate (%)
1	100	100
2	100	100
3	100	100
4	96	96

The *in vitro* release results of drug-loaded microneedles are shown in **Figure 3D**. Because the microneedle carrier material is a soluble polymer material, the cumulative release percentage reaches 64.96% within 30 minutes of the release, which is characterized by rapid release.

### 3.3 Effect of AM-ae on serum indexes of MGH rats

**Figure 4:**
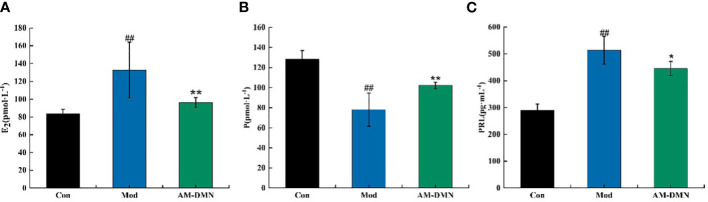
Effect of AM-ae on serum indexes of MGH rats **(A)**. Effect of AM-ae on E_2_ of MGH rats, **(B)** Effect of AM-ae on P of MGH rats, **(C)** Effect of AM-ae on PRL of MGH rats).

As shown in **Figure 4**, after modeling, compared with the Con group, the serum levels of E_2_ and PRL in the Mod group rats were highly significantly increased (*p*<0.01), and P levels were highly significantly decreased (*p*<0.01). After treatment with AM-DMN, compared with the Mod group, the E_2_ content in the serum of rats in the AM-DMN group was significantly decreased (*p*<0.01), the PRL content was significantly decreased (*p*<0.05), and the P content was significantly increased (*p*<0.01).

### 3.4 Evaluation of the quality of sequencing data of intestinal contents microbiota

**Figure 5:**
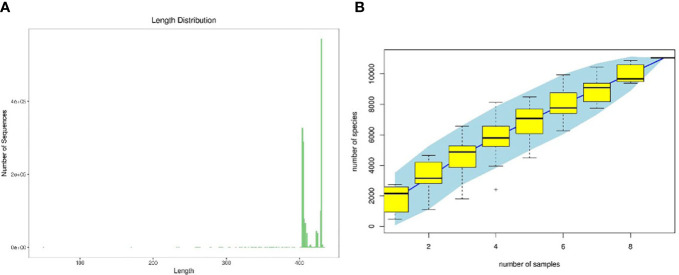
Quality evaluations of sequencing data of rat intestinal contents microbiota **(A)**. sequence length distribution diagram, **(B)** species accumulation curve diagram).

As shown in **Figure 5A**, according to the sequence length data derived from this sequencing, each sample’s sequence length was primarily 400–500 bp. **Figure 5B** illustrates as sample size rose, the number of OTUs climbed more slowly and then flattened out, showing that the total number of OTUs barely increased when more samples were added. This demonstrated that the samples used in this investigation were adequate to suit the needs of the study.

### 3.5 Effect of AM-ae on intestinal OTU, abundance grade, and alpha diversity in MGH rats

**Figure 6:**
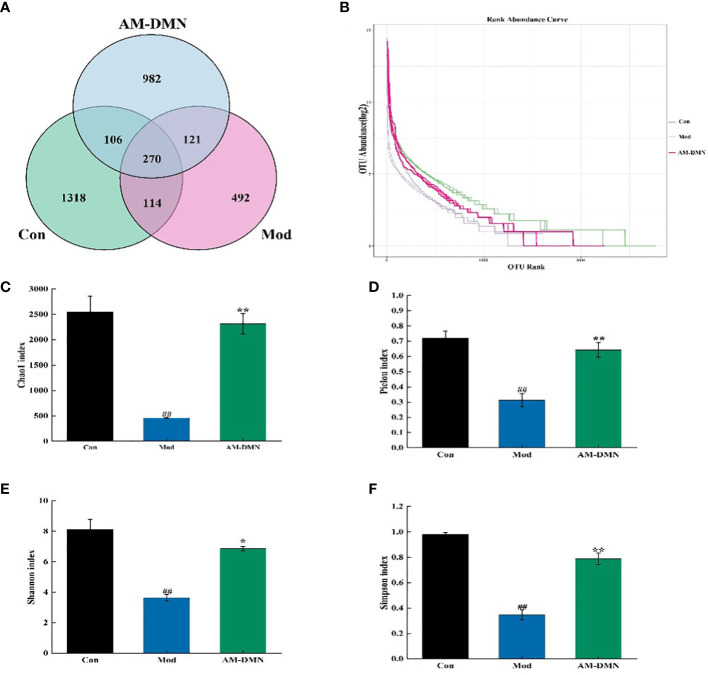
Effect of AM-ae on the number of intestinal OUT and alpha diversity of MGH rats **(A)**. number of intestinal OUT of rats, **(B)** Abundance grade curve, **(C)** Chao1 index, **(D)** Pielou index, **(E)** Shannon index, **(F)** Simpson index).

**Figure 6A** displays the OTU analysis. There was 1318, 492, and 982 OTUs total in the Con, Mod, and AM-DMN groups, respectively. There were 270 OTUs in the junction of the three groups. OTUs were considerably lower in the Mod group compared to the Con group, suggesting that MGH illness can cause rats’ gut microbiota to become unbalanced and to become less numerous. following AM-DMN therapy, the OTU count increased, but in the gut remained lower than in the Con group. Based on abundance log2 values, the rank-abundance distribution curve was drawn (**Figure 6B**). According to our findings, the Con group contained the most OTUs, which is in line with the Venn diagram previously mentioned. Alpha diversity refers to the diversity within a specific area or ecosystem and is a comprehensive indicator reflecting richness and evenness. Chao1 and Pielou indices are used to evaluate richness, and the larger its values, the more abundant the total number of species in the environment. As two other indicators for assessing diversity, the Shannon and Simpson index, the higher the value, the higher the diversity of species in the environment. As can be seen from **Figures 6C-F**, compared with the Con group, the Chao1, Pielou, Shannon, and Simpson in the intestinal flora of Mod rats were highly significantly decreased (*p*<0.01), it was demonstrated that MGH decreased the rat gut microbiota’s diversity and abundance. The Chao 1 index (*p*<0.01), Pielou index (*p*<0.05), Shannon index (*p*<0.01), and Simpson index (*p <*0.01) in the rat microbiota were considerably greater in the AM-DMN group compared to the Mod group, demonstrating that AM-DMN can increase the quantity and diversity of the intestinal microbiota in MGH rats.

### 3.6 Effect of AM-ae on intestinal Beta diversity in MGH rats

**Figure 7:**
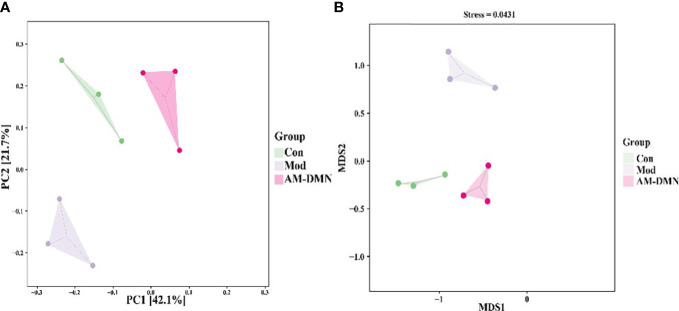
Effect of AM-ae on intestinal beta diversity of MGH rats **(A)**. PCA, **(B)** NMDS).

Beta diversity is also known as inter-habitat diversity. It is often used to study the relationship of species diversity between communities or the differences between samples. Its research methods include PCA and NMDS. The PCA diagram (**Figure 7A**) and NMDS diagram (**Figure 7B**) show that the distance between the intestinal microflora structure of the Mod group and Con group is significantly different, indicating that MGH can change the intestinal microflora structure of rats.

Compared with the Mod group, after AM-DMN treatment, the community structure similarity between the AM-DMN group and Con group is higher and relatively concentrated. β Diversity analysis showed that AM-ae could repair the bacterial structure of intestinal mucosa and restore it to normal.

### 3.7 Effect of AM-ae on the relative abundance of intestinal microflora in MGH rats

**Figure 8:**
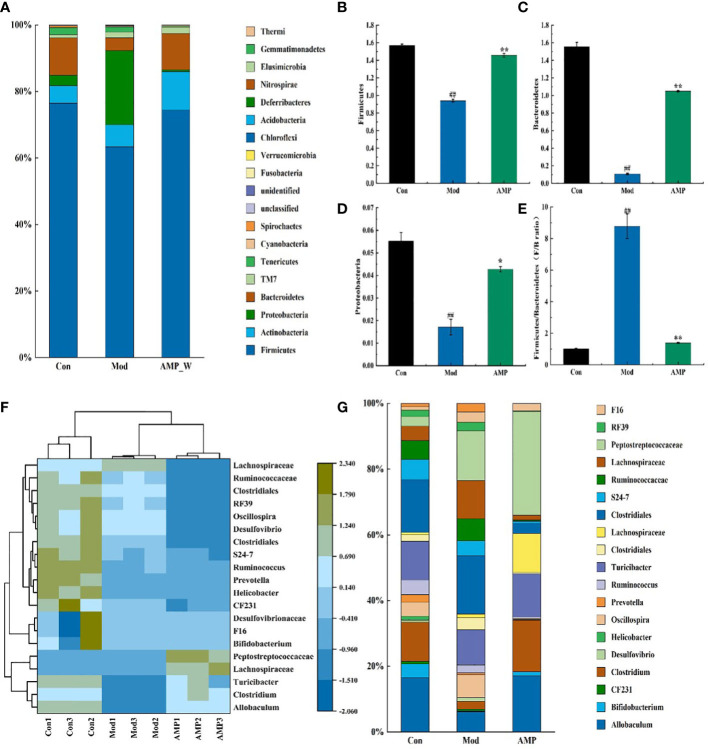
Effect of AM-ae on the relative abundance of intestinal microbiota in MGH rats **(A)**. Relative abundance of door level, **(B)** Firmicutes, **(C)** Bacteroidetes, **(D)** Proteobacteres, **(E)** Firmitutes/Bacteroides, **(F)** Horizontal relative abundance heat map, **(G)** Relative abundance at genus level).

The relative abundance of intestinal mucosal bacteria at the phylum level in rats was shown in **Figure 8A**, in which four taxa, Firmicutes, Bacteroidetes, Actinobacteria, and Proteobacteria were the dominant phylum, accounting for a larger proportion of the total microbiota, about 95%. As shown in **Figure 8B-D**, compared with the Con group, in the Mod group the relative abundance of Firmicutes, Bacteroidetes, and Proteobacteria decreased highly significantly (*p*<0.01). In the AM-DMN group compared with the Mod group, the relative abundance of Firmicutes and Bacteroidetes increased highly significantly (*p*<0.01), and the Proteobacteres increased significantly (*p*<0.05).

**Figure 8E** shows that the F/B ratio of Firmicutes and Bacteroides in the Mod group is significantly higher (*p*<0.01), but the F/B ratio was significantly decreased after AM-DMN treatment (*p*<0.01). The horizontal heat map of rat intestinal flora is shown in **Figure 8F**. The heat map can simultaneously reflect the information on species composition and abundance of the community, and visually reflect the differences and similarities of the composition of different samples or sub-groups of communities through color changes. At the same time, cluster analysis is carried out according to the similarity of species or samples. Among the tested genera, Con and AM-DMN can be well clustered into one group, while the Mod group is relatively scattered, which may be due to the differences in the changes of intestinal flora in rats caused by MGH. It can be seen from **Figure 8G** that Allobaculum, Clostridiales, S24-7, and Ruminococcaceae, etc. are the dominant genera at the genus level. Compared with the Con group, the relative abundance of Actinobacteria, Clostridium, S24-7, and Ruminoccaceae in intestinal flora of rats in the Mod group was significantly lower (*p*<0.01); Compared with the Mod group, the relative abundances of Allobaculum, Clostriales, S24-7 and Ruminoccaceae in AM-DMN group were significantly higher (*p*<0.01). It was confirmed that AM-ae could improve the disturbance of intestinal flora caused by MGH.

## 4 Discussion

Skin is the main barrier to transdermal drug delivery, which is composed of the epidermis, dermis, subcutaneous tissue, sebaceous glands, and sweat glands. Among them, the cuticle is the largest drug administration barrier. It is a special lipid, which has a strong barrier effect on both hydrophilic and lipophilic compounds, resulting in low drug permeability and unsatisfactory therapeutic effect. Therefore, to improve the barrier effect of cuticles on hydrophilic and lipophilic drugs, chemical and physical methods are usually used to improve the drug’s skin permeability (19). The chemical method is to add absorption enhancers into the prescription, which can improve the permeability of drugs on the skin surface. However, excellent absorption enhancers need to meet many conditions, such as non-toxicity, non-irritant and non-allergic and have good compatibility with drugs and other excipients, so it is difficult and limited to select the appropriate absorption enhancers. Microneedles are one of the physical transdermal drug delivery methods, which can puncture the cuticle and deliver drugs to the skin, improving the permeability and bioavailability, Besides, the drug delivery process is safe and non-irritating to the skin, which is a painless and minimal invasive drug delivery method (20, 21). In this study, DMN was prepared from a biodegradable polymer material, which has the characteristics of biological stability, non-immunogenicity, non-toxicity, and no irritation to the human body. According to relevant literature reports, DMN is often used as a carrier for drug delivery such as insulin, 5-aminolevulinic acid, low molecular weight heparin, ovalbumin, adenovirus vector, a variety of vaccine antigens, and biomolecular molecules (22). It has been used in the treatment of skin diseases (23–25), metabolic diseases (26, 27), immune diseases (2, 28), and medical cosmetology (29, 30). The results of this study showed that the construction of soluble microneedles based on AM-ae has a good therapeutic effect on the treatment of breast hyperplasia.

MGH is a pathological hyperplasia of the breast lobule caused by an imbalance between estrogen and progesterone, when the E_2_ level is excessive or the P level is too low *in vivo*, may lead to incomplete epithelial differentiation of the proliferating glands and non-regeneration of proliferative tissues, leading to MGH (31). In mammals, as a cytokine, PRL can regulate mammary gland development, promote milk secretion, and affect milk protein synthesis, while excessive PRL can cause structure disorder of the mammary gland (32). Sex hormones affect the intestinal microbiota by regulating intestinal barrier permeability and integrity, sex hormone receptors, β-glucuronidase, bile acids, intestinal immunity, etc. At the same time, intestinal flora also affects the secretion of sex hormones. Yi Wu et al. demonstrated that sex hormones may be involved in sexual dimorphism in bile acid metabolism by regulating the abundance of these bacteria (33). *Atractylodis Macrocephalae Rhizoma*, a dried rhizome of Atractylodes macrocephala Koidz., a plant of the composite family, has a long history of medicinal use in China. It is mainly used for deficiency of the spleen, lack of food, abdominal distension and diarrhea, phlegm, dizziness and palpitation, edema, spontaneous sweating, fetal movement, etc. The pharmacological effects of *Atractylodis Macrocephalae Rhizoma* are mainly in the gastrointestinal system, immune system, and urinary system. It has the functions of anti-aging, enhancing immunity, anti-tumor, anti-inflammatory, regulating gastrointestinal function, and regulating water and salt metabolism (34). According to the results of pharmacological experiments, AM-DMN can significantly reduce the content of E_2_ and PRL, and increase the contents of P, which has the effect of treating MGH. Some studies have shown that intestinal flora plays an important role in estrogen metabolism because intestinal flora can affect the enterohepatic circulation and reabsorption of estrogen. Estrogen and its metabolites can be excreted from bile glycolaldehyde or sulfonation. According to radioactive element labeling, about 65% of estradiol is excreted through bile, and the estradiol reabsorption process occurs when estrogen is excreted in the bile unclotted by β-glucuronidase in the gut, resulting in its reabsorption into circulation (35, 36). Shimizu K et al. found that adding intestinal flora of normal mice could normalize the estrous cycle of sterile female mice with reproductive impairment (37). The results of 16s rRNA showed that after administration of AM-DMN, the abundance, and diversity of intestinal flora in MGH mice increased, the relative abundance of dominant bacteria Firmicutes and Bacteroidetes increased, the F/B value decreased, and the resorption of estrogen decreased. Based on this, we speculated that the effect of AM-DMN on MGH mice may be related to the regulation of intestinal flora composition and abundance, but the specific mechanism still needs to be further explored. In conclusion, the prepared AM-DMN has a complete microneedle array, and the performance test results meet the requirements, which can improve intestinal flora and hormone disturbance induced by MGH through topical application.

## Data availability statement

The datasets presented in this study can be found here: doi: 10.5061/dryad.79cnp5j0h.

## Ethics statement

All experimental procedures involving animals were approved by the Animal Ethics Committee of the Animal Experimental Center of Jiamusi University.

## Author contributions

YP prepared the drafting of the manuscript and interpretation of data. QG and YLW analyzed in the analyzing of part of the data. CXL, YW, SL, MJQ, LQZ, ALT and YT performed the experiment. HZ designed the study and revised the manuscript. All authors contributed to the manuscript revision, read, and approved the submitted version.
